# Memories of the Future: New Insights into the Adaptive Value of Episodic Memory

**DOI:** 10.3389/fnbeh.2013.00047

**Published:** 2013-05-23

**Authors:** Karl K. Szpunar, Donna Rose Addis, Victoria C. McLelland, Daniel L. Schacter

**Affiliations:** ^1^Department of Psychology, Harvard UniversityCambridge, MA, USA; ^2^Center for Brain Science, Harvard UniversityCambridge, MA, USA; ^3^School of Psychology, The University of AucklandAuckland, New Zealand; ^4^Centre for Brain Research, The University of AucklandAuckland, New Zealand

Ingvar (1979, p. 21) theorized that memory plays a key role in allowing individuals to construct “alternative hypothetical behavior patterns in order to be ready for what may happen,” a process that he characterized as a “simulation of behavior”. Several years later, Ingvar elaborated these ideas by observing that “concepts about the future, like memories of past events, can be remembered, often in great detail” (Ingvar, 1985, p. 128), and that such “memories of the future” may offer important insights into the adaptive nature of human cognition. For instance, although the ability to simulate alternative versions of the future can benefit behavior, at least part of the adaptive advantage of future thinking may depend on the ability to remember the contents of simulated events (for discussion, see Suddendorf and Corballis, 1997, 2007; Szpunar, 2010; Schacter, 2012; Schacter et al., 2012). As an example, enlisting mental simulations of the future to help determine the best approach for resolving a conflict at home or in the workplace may confer few advantages if the outcome of the simulation process is not remembered at the time the simulated behavior is executed. Although much research exists concerning prospective memory – remembering to carry out future intentions (e.g., Kliegel et al., 2008; see also Brewer and Marsh, 2009) – there is a striking lack of evidence concerning “memory of the future” in the sense discussed by Ingvar, that is, memory for the contents of future simulations. Recently, however, several studies have provided the first glimpses into how well people remember details associated with simulated future events. In particular, these studies have demonstrated (1) enhanced memory for event details that are encoded with the future in mind, (2) factors that may influence the retention of simulated future events over extended time periods, and (3) neural correlates associated with encoding simulated future events. Next, we provide a brief overview of this emerging line of research, underscore the significance of various findings along with suggestions for future research directions, and conclude by discussing the relevance of this work to the concept of episodic memory. As we expand on below, it is our opinion that these new studies represent not only a useful extension of Ingvar's (1979, 1985) seminal observations, but that they also offer novel insights into the adaptive value of episodic memory.

## Future-Oriented Encoding Processes Enhance Retention

Klein et al. (2012, p. 240) have recently noted that systems of memory, including episodic memory, may be “designed by evolution to interface with systems of long-term planning” (see also Suddendorf and Corballis, 2007). Accordingly, memory systems should be more efficient in dealing with encoding manipulations that encourage future-oriented processes such as planning than other well-established encoding manipulations that are not necessarily oriented toward the future (e.g., imagery, self-relevance; see also Klein et al., 2002; Klein et al., 2010; McDonough and Gallo, 2010). A recent line of research supports this prediction. Klein et al. (2010) asked separate groups of participants to think about one of three camping scenarios: imagining a future camping trip, remembering a past camping trip, or imagining a typical campsite without reference to the past or future. During the simulation/retrieval task, participants were asked to indicate how likely it was that various items (e.g., tent, rope) were incorporated into their self-generated scenarios. After a brief delay, participants were presented with a blank sheet of paper and asked to recall the list of items associated with the simulation/retrieval task. Participants who had simulated a future camping trip remembered more items than participants who had remembered a camping trip or simulated a typical campsite in an atemporal manner.

Notably, Klein et al. (2010) included a fourth encoding condition that asked a separate group of participants to imagine a hypothetical camping scenario in which they were stranded in a forest, and to indicate how likely it was that the same set of items (e.g., tent, rope) would enhance their chances of survival. This latter condition was included in response to a recent series of studies by Nairne and colleagues demonstrating that attending to the survival value of to-be-encoded information also enhances retention relative to other well-established encoding manipulations (for a review, see Nairne, 2010). Klein et al. (2010) hypothesized that survival processing may enhance retention to the extent that it calls upon future-oriented processes such as planning. Along these lines, Klein et al. (2010) further suggested that the survival scenario might evoke planning in some participants but not others; for instance, some participants may think about the survival scenario in an atemporal manner. Hence, while details associated with the survival scenario should be better remembered than details associated with scenarios that do not evoke planning (e.g., remembering a camping trip), details associated with the survival scenario should not be remembered as well as details associated with scenarios that always evoke planning (e.g., imagining a future camping trip). Klein et al. (2010) found support for both of these predictions. In a subsequent study, Klein et al. (2011) systematically varied the involvement of planning and survival processing in various simulated scenarios, and found that participants were better able to remember details associated with scenarios that had evoked planning, whether or not survival processing was relevant, than details associated with survival scenarios that had not evoked planning.

Nonetheless, some studies have failed to demonstrate a mnemonic advantage for encoding conditions that foster planning as compared to survival processing (e.g., Kang et al., 2008; Weinstein et al., 2008). In response to these incongruent findings, Klein et al. (2010, 2011) pointed out that enhanced memory for plans might depend on the extent to which plans evoke concepts of the personal future, such as planning for events that participants have experienced in the past and expect to experience again in the future. To test this claim, Klein et al. (2012) asked separate groups of participants to plan for familiar (e.g., dinner party) and unfamiliar (e.g., trip to Antarctica) scenarios. Participants who had made plans for familiar scenarios remembered more details associated with those scenarios than participants who had made plans for unfamiliar scenarios.

Hence, the available evidence suggests that people are rather adept at encoding and subsequently remembering details about simulated future events. We conclude this section by discussing a recent study demonstrating how enhanced memory for details associated with future events can be used to help people distinguish between memories of the past and future. Earlier research on the relation between memory and imagination had demonstrated that memories and simulated events are best characterized by distinct phenomenological features: remembered events tend to be characterized by enhanced perceptual clarity whereas imagined events tend to be characterized by increased representation of cognitive operations (for a review, see Johnson, 1988). Building on the results of Johnson and others, McDonough and Gallo (2010) found that people were able to make use of phenomenological characteristics such as perceptual clarity and cognitive operations when discriminating between memories of past and future events. Interestingly, the authors also found that the distinguishing characteristics of future events – cognitive operations – were more diagnostic in discriminating between memories of past and future events than were the distinguishing characteristics of memories – perceptual clarity. Accordingly, McDonough and Gallo (2010) noted that this latter pattern of results might reflect the workings of a memory system whose primary function involves “imagination and planning for future events” (p. 7).

In sum, the results of a number of studies have demonstrated that encoding manipulations that encourage future-oriented processes such as planning improve retention relative to other well-established encoding manipulations. Moving forward, studies that can identify the possible factors that underlie the mnemonic advantage associated with future-oriented encoding processes, such as the role of familiarity and personal experience (Klein et al., 2012), will be of considerable interest. As an initial step in that direction, McLelland et al. (submitted) identified several event characteristics that predicted the likelihood of successful encoding of future events. Imagined future events that were rated by participants as being more detailed, more plausible, and involving more familiar elements (in particular, events with more familiar people) were all significantly more likely to be later remembered than imagined events with low ratings on such characteristics.

## Retention of Simulated Future Events Over Extended Periods

In the studies discussed thus far, memory for future simulations was tested after relatively brief study-test delays (usually 5–20 min post simulation). Yet in the everyday situations discussed by Ingvar (1985), it may be necessary to retain a simulation over days and weeks. However, only two studies of which we are aware have examined memory for simulated future events at extended delays. In one study, Szpunar et al. (2012) asked participants to imagine future scenarios comprising people, locations, and objects that had been extracted from autobiographical memories or participant-generated lists. Memory for simulations was tested either shortly after simulation (20 min) or after an extended delay (24 h) by providing two elements of the simulated episode (e.g., person and object) and probing for recall of the third element (e.g., location). Interestingly, more emotional simulations (i.e., positive and negative events) were remembered than neutral simulations after the short delay, whereas more positive and neutral simulations were remembered than negative simulations after the long delay. That is, negative simulations were forgotten more quickly than positive and neutral simulations. This pattern of data may reflect the influence of fading affect bias (Walker and Skowronski, 2009), such that the affect that binds event details together (Mather and Sutherland, 2011) may dissipate more quickly for negative than positive events. Notably, Gallo et al. (2011) also found a positivity bias in memory for simulated future events after 24 h in young and old adults. Hence, the available data suggests that people may remember a rosy simulated future. Further studies of memory for simulated emotional events may thus have the potential to provide insights into various mood disorders such as anxiety and depression.

## Neural Substrates for Encoding Simulations of Future Events

So far, studies concerning “memory of the future” have focused mainly on cognitive processes. Although little is known about the neural processes that support encoding of simulated future events, a recent study by Martin et al. (2011) indicates that the hippocampus plays an important role. During fMRI scanning, participants imagined future scenarios comprising people, locations, and objects that were extracted from autobiographical memories. Memory for simulations was tested shortly after the scan by providing two elements of the simulated episode (e.g., person and object) and probing for recall of the third element (e.g., location); a simulation was classified as “remembered” when participants recalled the third element correctly, and “forgotten” when they did not. Greater hippocampal activity was observed during construction of subsequently remembered than forgotten simulations even when controlling for the amount of detail associated with each simulation (for discussion of related findings, see Addis and Schacter, 2012; Schacter et al., 2012). Looking ahead, studies will be needed that can isolate the neural circuitry that underlies the mnemonic advantage produced by encoding information with the future in mind.

## Summary and Conclusions

Over the last four decades, the concept of episodic memory has evolved into a multifaceted construct that is of great interest to researchers in various areas of psychology and neuroscience (for recent overviews, see Szpunar and McDermott, 2008; Tulving and Szpunar, 2009). Here, we have highlighted recent insights into an adaptive feature of episodic memory first noted by Ingvar (1985): “memories of the future,” like memories of past events, can help to guide behavior. Given the potential theoretical and practical importance of understanding memories of the future, it is perhaps surprising that so little relevant experimental work has been done. Developing a more in depth understanding of why simulated future events are well remembered, what factors influence the long-term retention of simulated future events, and how the brain supports the encoding of simulated future events, represent important avenues for future research that are likely to broaden our understanding of the utility of episodic memory in everyday life.
